# Challenges for room temperature operation of electrically pumped GeSn lasers

**DOI:** 10.1038/s41598-024-60686-3

**Published:** 2024-05-05

**Authors:** A. R. Ellis, D. A. Duffy, I. P. Marko, S. Acharya, W. Du, S. Q-. Yu, S. J. Sweeney

**Affiliations:** 1 James Watt School of Engineering, University of Glasgow, Glasgow, G12 8LT UK; 2 Material Science and Engineering Program, University of Arkansas, Fayetteville, AR 72701 USA; 3 Department of Electrical Engineering and Computer Science, University of Arkansas, Fayetteville, AR 72701 USA; 4 Institute for Nanoscience and Engineering, University of Arkansas, Fayetteville, AR 72701 USA

**Keywords:** Semiconductor lasers, Electronic devices

## Abstract

Recent demonstrations of room-temperature lasing in optically pumped GeSn show promise for future CMOS compatible lasers for Si-photonics applications. However, challenges remain for electrically pumped devices. Investigation of the processes that limit device performance is therefore vital in aiding the production of future commercial devices. In this work, a combined experimental and modelling approach is utilised to explore the dominant loss processes in current devices. By manipulating the band structure of functioning devices using high hydrostatic pressure techniques at low temperature, the dominant carrier recombination pathways are identified. This reveals that 93$~\pm ~$5% of the threshold current is attributable to defect-related recombination at a temperature, T = 85 K. Furthermore, carrier occupation of L-valley states (carrier leakage) is responsible for 1.1$~\pm ~$0.3% of the threshold current, but this sharply increases to 50% with a decrease of just 30 meV in the L-$\Gamma$ separation energy. This indicates that thermal broadening of a similar order may reproduce these adverse effects, limiting device performance at higher temperatures. Temperature dependent calculations show that carrier occupation of indirect valley L-states strongly affects the transparency carrier density and is therefore very sensitive to the Sn composition, leading to an effective operational temperature range for given Sn compositions and strain values. Recommendations for future device designs are proposed based on band structure and growth optimisations.

## Introduction

Progress in Si-photonics has expedited dramatically over the past decade due to significant developments in both active and passive, CMOS-compatible components, such as high-speed modulators^1,2^, photodetectors^3^ and multiplexers^4^. However, the lack of CMOS-compatible light sources has thus far limited the feasibility of fully monolithic optoelectronic integrated circuits (OEICs). Whilst a number of novel approaches for integration of III-V lasers onto a Si platform have yielded high-performance devices, such techniques come with large costs and limited scalability due to the challenging processing required for fabrication^5^ or issues with material quality^6^. Thus, an electrically pumped laser that can exploit mature CMOS foundries and processes remains a key aim for achieving cost-effective, small-footprint OEICs.

GeSn semiconductor alloys have emerged as a promising option for realising such devices due to its transition to a direct gap material at relatively low Sn fractions. This has recently been shown to be a smooth transition between indirect and direct bandgap character, which occurs over the 6-10% Sn range in unstrained material^7^. The first GeSn laser was realised by Wirths et al. in 2015, operating under optical pumping up to a temperature of 90 K^8^. Since then, significant improvements in the performance of optically pumped devices have been achieved in microdisks^9^, ridge-waveguides^10^ and bridges^11^ which has led to room-temperature (RT) lasing. Quantum well devices^12^ and lasers operating in CW^13^ have also been reported at lower temperatures. The first electrically pumped device was produced in 2020, consisting of a Fabry-Pérot bulk double heterostructure operating up to 100 K under pulsed excitation^14^. Electrically pumped devices of differing design geometries have also been recently achieved, operating below 90 K^15^.

The heightened operational temperature observed in optically pumped devices is typically the result of high levels of tensile strain or high Sn fractions, both of which result in increased splitting between the $\Gamma$ and L-valleys. This energetic separation is commonly referred to as the directness of the material, denoted by ${\Delta \textrm{E}_{\textrm{L}-\Gamma }}$. The design considerations for production of electrically pumped devices are vastly more complex than optically pumped structures. These necessary complications often introduce a large number of pathways for loss, such as non-radiative recombination via trap-states in defective buffer layers, insufficient carrier confinement between layers and absorption in metallic contacts. Some of these concerns have been addressed in more recent studies, raising the operational temperature to 110 K^16^, however further information about carrier dynamics within such structures is required to attain the significant performance improvements needed for commercial applications.

Recombination processes can be probed by subjecting the laser to high hydrostatic pressures. This causes a reversible increase in the $\Gamma$ and L bandgaps by amounts determined by the pressure coefficients, $\textrm{dE}_{\Gamma }/\textrm{dP}$ and $\textrm{dE}_{L}/\textrm{dP}$, whilst ensuring key crystal symmetries and heterostructure offsets are maintained. This enables the variation in bandgap dependent recombination mechanisms to be measured, and makes it uniquely suited to probing materials with low levels of directness.

In this paper, we investigate the dominant carrier recombination processes in first generation, electrically pumped GeSn double heterostructure lasers using high-hydrostatic pressure measurements at low temperature. A theoretical study on the effect of the active region directness on the thermal performance of bulk GeSn devices is subsequently conducted through calculations of the material gain, utilising an 8-band k$\cdot$p model to account for band non-parabolicities. The design space for bulk GeSn lasers is discussed with respect to optimising material gain and minimising optical losses through band structure engineering.

## Results

### High-hydrostatic pressure measurements

The devices investigated during this study are GeSn/SiGeSn double heterostructure laser diodes grown on an industry standard 200 mm Si(100) substrate using chemical vapour deposition (CVD). The structure consists of five distinct layers, from bottom to top; a fully relaxed, 500 nm Ge buffer (n-doped - 1$\times$10$^{19}$ cm$^{-3}$), a 700 nm GeSn buffer with Sn gradient ranging between 8 % (bottom) and 10.5 % (top), a 1$~{\mu }$m thick GeSn$_{0.105}$ active region, a 170 nm Si$_{0.03}$Ge$_{0.89}$Sn$_{0.08}$ capping layer (p-doped - 1$\times$10$^{18}$ cm$^{-3}$), and a 70 nm Si$_{0.03}$Ge$_{0.89}$Sn$_{0.08}$ Ohmic contact (p-doped - 1$\times$10$^{19}$ cm$^{-3}$). Using C-V and Hall test samples, the background p-doping of the active region at 300 K was estimated to be 1$\times$10$^{17}$cm$^{-3}$. Devices were fabricated into ridge waveguide structures with stripe widths of 100 ${\mu }$m and a 1.7 mm cavity length, with Cr/Au *p* and *n* electrodes, resulting in electrically pumped GeSn laser diode devices. Further growth information can be found in reference^14^.

**Figure 1:**
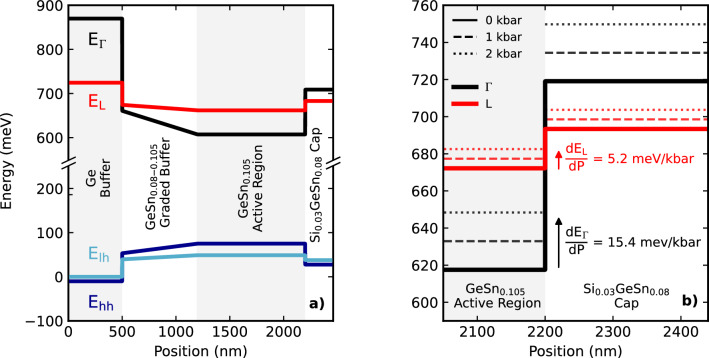
(**a**) Heterostructure band edge diagram (unbiased, at 85 K, atmospheric pressure) illustrating confinement of electrons by the SiGeSn capping layer and hole confinement by a graded GeSn buffer layer. (**b**) shows the conduction band edges modelled at 0 (solid), 1 (dashed) and 2 kbar (dotted) for the $\Gamma$- (black) and L-valleys (red), showing similar pressure coefficients for like valleys in the cap and active layers.

Following wire bonding and header mounting, devices were loaded into a copper-beryllium cell and connected to a UniPress U11 helium gas compressor system^17,18^.The cell was housed within a closed-cycle cryostat, enabling high pressure measurements to be conducted at temperatures below the 100 K operating temperature of the devices. Square voltage pulses of 1 ${\mu }$s were supplied by an AVTECH 1011B1 pulse generator at a frequency of 1 kHz to minimise internal heating effects. Emission was collected as a function of increasing injection current through a sapphire window in the cell and focused onto a liquid nitrogen cooled InSb detector connected to a Signal Recovery 7265 DSP lock-in amplifier. Light output-current (LI) curves were collected for pressures between 0 and 2 kbar at a fixed temperature of 85 K, from which the threshold current densities were extracted. The flat band alignment at atmospheric pressure and 85 K is shown in Fig. 1a, while Fig. 1b shows the effect of increasing pressure on the conduction band edges of the active region and cap layers.

The threshold current density is dependent on the injection efficiency and the rate of carrier depletion from the direct valleys. The processes responsible for reducing the number of carriers in these valleys can be categorised generally as radiative and non-radiative recombination, and carrier leakage. In the low temperature regime of interest in this study where Auger recombination is expected to be small, the total current density at threshold then takes the form

1 $$\begin{aligned} J_{th} = J_{th}^{rad} + J_{th}^{def} + J_{th}^{lk}, \end{aligned}$$

where $J_{th}^{rad}$ and $J_{th}^{def}$ are the current densities at threshold associated with radiative recombination and non-radiative defect-related recombination, respectively. $J_{th}^{lk}$ accounts for processes where injected carriers occupy indirect states in the active region or leak to states outside of the active region. Knowledge of the pressure dependence of the current density for each of these processes therefore enables their fractional contributions to total threshold to be determined.

The radiative current in bulk materials is proportional to $E_{g,\Gamma }^{5/2}$, where $E_{g,\Gamma }$ is the direct bandgap in the lasing medium^19^. The leakage current for carriers to L-valley states in the active region or cap layer can be approximated by the simple expression $J = J_0\exp (-E_a/k_BT)$ where $E_a = E_L - E_{fc} \approx E_L - E_{\Gamma }$. Such expressions were previously shown to be a valid approximation for L-^20^ and X-valley^21^ leakage in III-V based systems. Here $E_L$ and $E_{\Gamma }$ represent the conduction band minima of each valley, and $E_{fc}$ is the conduction band quasi-Fermi energy. Due to the proximity of the L-valleys in the cap and active region, an average L-valley energy is utilised in the calculations. The pressure dependence of this contribution is then $J^{lk}(P) \propto \exp (-dE_a/dP \cdot P/k_B T)$^22^. Since the pressure coefficients of like-valleys are similar for group IV materials^7,23^, as indicated in Fig. 1b, leakage into the $\Gamma$-minima of the cap material is expected to be pressure independent as the large $\Gamma$ valley offset between the active region and cap layer is maintained. Recombination via defects is not expected to have a strong pressure dependence^24^.

**Figure 2:**
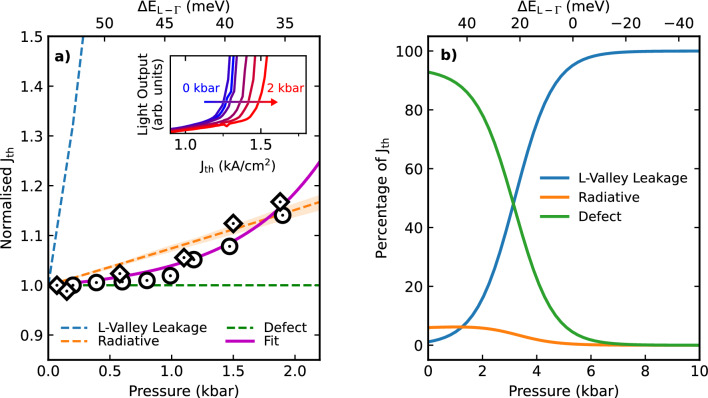
(**a**) The threshold current as a function of pressure (normalised to atmospheric pressure), where diamond and circular markers illustrate two devices with the same heterostructure. The blue, green and orange dashed lines show the pressure dependencies of the leakage, defect and radiative recombination processes, with the radiative uncertainty indicated by the shaded region. The top axis denotes the modelled levels of directness in the active region material. A subset of the LI curves with increasing pressure used to determine threshold are shown in the inset. (**b**) The variation in fractional contributions of each process to the total threshold as a function of pressure, extrapolated from the fit in (**a**).

To calculate each of these relations, the partially relaxed band edges of Fig. 1 were computed as a function of pressure. Values for most parameters used in the model were determined through linear interpolation of the constituent elements. However, the direct and indirect bandgaps were calculated using

2 $$\begin{aligned} E^{i}_{g,SiGeSn} = xE^{i}_{g,Si}+ yE^{i}_{g,Ge} + zE^{i}_{g,Sn} - xyb^{i}_{SiGe} - yzb^{i}_{GeSn} - xzb^{i}_{SiSn}, \end{aligned}$$

to account for the inclusion of bowing terms for multinary alloys. Here the first three terms denote the linear interpolation of the bandgaps of the constituent elements, and *b* are the bowing parameters of the constituent binaries. *x*, *y* and *z* denote the fractions of Si, Ge and Sn in the material, respectively, and *i* denotes either the L or $\Gamma$ valleys. Values for the bowing parameter of the direct valleys for GeSn quoted in literature span a range of 1.94–2.92 eV^25,26^, whilst a number of groups have suggested that a further non-linear term or compositional dependence may be required to accurately replicate the band structure^27^. Our studies indicate that the choice of bowing parameter, within the range permitted by our experimental data, has a minimal effect on the presented results. We adopt the temperature-dependent bowing terms for GeSn given by, $b_{GeSn}^{\Gamma } = 2.55 - 4\times 10^{-4} T$ and $b_{GeSn}^{L} = 0.89 - 7\times 10^{-4} T$^28^. The bowing parameters for SiGe are taken as the standard $b_{SiGe}^{\Gamma } = 0.21$ eV and $b_{SiGe}^{L} = 0.335$ eV^29^. The uncertainty in the bowing parameters for SiSn is significantly higher, with literature values for the ${\Gamma }$-valley spanning from −21 eV^30^ to 24 eV^31^. The low Si and Sn fractions in the SiGeSn layers modelled in this work are such that the effect on the overall band structure is minimal. Therefore, values of $b_{SiSn}^{\Gamma } = 2.124$ eV and $b_{SiSn}^{L} = 3.915$ eV are also taken from^29^ for consistency.

The calculated pressure dependence of each mechanism is illustrated by a dashed line in Fig. 2a. The uncertainty in $b^{\Gamma }_{GeSn}$ only affects the trend of the radiative current, and is illustrated by the shaded orange region. A subset of the collected LI curves as a function of pressure are displayed as an inset in Fig. 2, with the extracted threshold current values of two devices from the same wafer denoted by circular and diamond markers in the main figure. The threshold shows little change within experimental uncertainty up to 1 kbar, before rising sharply between 1-2 kbar, corresponding to a decrease in directness of 10–20 meV. The produced fit to the measured data is achieved by weighting the normalised contributions of each recombination pathway, *i*, using

3 $$\begin{aligned} \frac{J_{th}(P)}{J_{th}(0)} = \sum _{i} C_{i}(0) \frac{J_{th, i}(P)}{J_{th, i}(0)}, \end{aligned}$$

where $C_i(0)$ are fitting factors that determine the fractional contribution of each process at atmospheric pressure. To reduce uncertainty in the fitting parameters, a minimum and maximum value for $J_{th,rad}(0)$ was calculated for structures with minimal and significant levels of optical loss, respectively. $C_{rad}(0)$ was then set to vary according to these constraints while the pressure insensitive and L-valley leakage terms were allowed to vary between 0-1, with the condition that $\sum _{i} C_i(0) = 1$.

The presented fit (magenta) shows an L-valley leakage fraction of 1.1 ± 0.3%, radiative recombination contributes 6$~\pm ~$4% and a pressure independent path accounts for 93 $\pm ~$5%, all at atmospheric pressure. It should be noted that the effect of the uncertainty in $b^{\Gamma }_{GeSn}$ on the resultant fit is too small to be visible on the plot. The large pressure insensitive contribution likely stems from non-radiative recombination in the buffer layer due to high threading dislocation densities^14^. However, at 85 K this reveals that L-valley leakage is impacting these devices. Fig. 2b depicts the fractional contributions of each process with increasing pressure, determined by extrapolating the fit to higher pressures. It can be seen that a decrease in active region directness as low as 30 meV can result in L-valley leakage currents (blue) comparable to that of the defect related current (green). A further decrease of 10 meV results in an L-valley leakage current 4$\times$ that of the defect related current. Thus, even a low to moderate decrease in the directness can result in a large increase in carriers escaping to indirect valleys in the active region and capping layer.

Similarly, at higher temperatures the fractional occupation of the indirect valleys will be increased as a result of thermal broadening. Carriers in these states have no route for efficient radiative recombination, and therefore act as a source of optical loss through free carrier absorption (FCA). Subsequent carrier relaxation results in device heating, further populating the L-valleys in a thermal runaway process^32^. Such optical losses are detrimental to device threshold and may contribute significantly towards limiting the maximum operational temperatures. Thus, as pressure measurements illustrate such a strong increase in carrier leakage with decreasing directness, it is important to analyse how thermal broadening impacts the carrier occupation of the indirect valleys and the impact this has on device performance.

### Temperature dependent modelling

**Figure 3:**
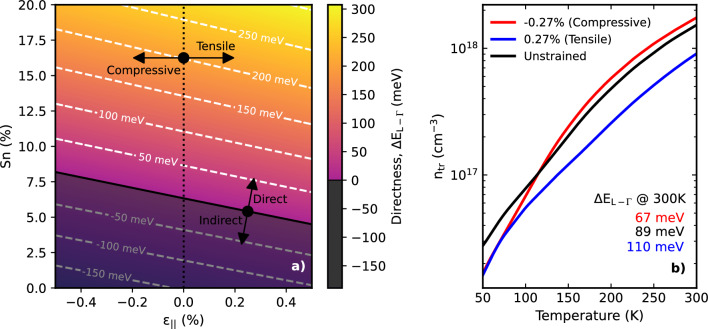
(**a**) The material directness ${\Delta \textrm{E}_{\textrm{L}-\Gamma }}$ and a function of Sn and attainable strains for bulk devices at 300 K. (**b**) The effect of small residual tensile (blue) and compressive (red) strains on the transparency carrier density in Ge$_{0.895}$Sn$_{0.105}$, compared to unstrained material (black), with the corresponding directness of each strain state included for comparison. Increasing tensile strain provides the best performance due to lifting of valence band degeneracy paired with increased directness.

In attempts to reduce leakage currents, studies thus far have focused primarily on improving barrier offsets through Si alloying, or utilising highly doped barriers to fill indirect states^33^. Although both approaches are able to provide minor improvements in the material gain, it is important to investigate the fundamental limitations placed on device performance by the bulk active region material. The material directness is known to increase with increasing Sn content and tensile strain, as shown at RT in Fig. 3a. We therefore wish to analyse how engineering these two variables within the achievable limits for bulk devices could improve performance. We probe this by calculating the bulk material gain as a function of both temperature and carrier injection for materials with varying Sn fractions, and for small levels of residual strain. To accurately account for band non-parabolicity, the electronic band structure is modelled using an 8-band k$\cdot$p Hamiltonian, from which the material gain is directly calculated. The unstrained Hamiltonian is given by^34^

4 $$\begin{aligned} H = \begin{pmatrix} ~E_{CB}^{GeSn}~ &{} ~~-\sqrt{3}T_+~~ &{} ~~\sqrt{2}U~~ &{} ~~-U~~ &{} ~~0~~ &{} ~~0~~ &{} ~~-T_-~~ &{} ~~-\sqrt{2}T_-~~\\ ~ &{} E_{HH}^{GeSn} &{} \sqrt{2}S &{} -S &{} 0 &{} 0 &{} -R &{} -\sqrt{2}R\\ ~ &{} ~ &{} E_{LH}^{GeSn} &{} Q &{} T^*_+ &{} R &{} 0 &{} \sqrt{3}S\\ ~ &{} ~ &{} ~ &{} E_{SO}^{GeSn} &{} \sqrt{2}T_+^* &{} \sqrt{2}R &{} -\sqrt{3}S &{} 0\\ ~ &{} ~ &{} ~ &{} ~ &{} E_{CB}^{GeSn} &{} -\sqrt{3}T_- &{} \sqrt{2}U &{} -U \\ ~ &{} ~ &{} ~ &{} ~ &{} ~ &{} E_{HH}^{GeSn} &{} \sqrt{2}S^* &{} -S^* \\ ~ &{} ~ &{} ~ &{} ~ &{} ~ &{} ~ &{} E_{LH}^{GeSn} &{} Q \\ ~ &{} ~ &{} ~ &{} ~ &{} ~ &{} ~ &{} ~ &{} E_{SO}^{GeSn} \end{pmatrix} \end{aligned}$$

where the matrix elements are

5 $$\begin{aligned} E_{CB}^{GeSn}({\textbf {k}})&= E_{CB}^{GeSn}(0) + \frac{\hbar ^2}{2m_0}s_C\big (k_{||}^2+k_z^2\big ), \nonumber \\ E_{HH}^{GeSn}({\textbf {k}})&= E_{HH}^{GeSn}(0) - \frac{\hbar ^2}{2m_0}\bigg [\big (\gamma _1 + \gamma _2\big )k_{||}^2 + \big (\gamma _1 - 2\gamma _2\big )k_{z}^2\bigg ], \nonumber \\ E_{LH}^{GeSn}({\textbf {k}})&= E_{LH}^{GeSn}(0) - \frac{\hbar ^2}{2m_0}\bigg [\big (\gamma _1 - \gamma _2\big )k_{||}^2 + \big (\gamma _1 + 2\gamma _2\big )k_{z}^2\bigg ], \nonumber \\ E_{SO}^{GeSn}({\textbf {k}})&= E_{SO}^{GeSn}(0) - \frac{\hbar ^2}{2m_0}\gamma _1\big (k_{||}^2+k_z^2\big ), \nonumber \\ T_\pm ({\textbf {k}})&= \frac{1}{\sqrt{6}}P\big (k_x\pm ik_y\big ), \nonumber \\ U({\textbf {k}})&= \frac{1}{\sqrt{3}}P k_z, \nonumber \\ S({\textbf {k}})&= \sqrt{\frac{3}{2}}\frac{\hbar ^2}{m_0}\gamma _3k_z\big (k_x-ik_y\big ), \nonumber \\ R({\textbf {k}})&= \frac{\sqrt{3}}{4}\frac{\hbar ^2}{m_0}\bigg [\big (\gamma _2+\gamma _3\big ) \big (k_x-ik_y\big )^2-\big (\gamma _3-\gamma _2\big )\big (k_x-ik_y\big )^2\bigg ],\nonumber \\ Q({\textbf {k}})&= -\frac{1}{\sqrt{2}}\frac{\hbar ^2}{m_0}\gamma _2k_{||}^2+\sqrt{2}\frac{\hbar ^2}{m_0}\gamma _2k_z^2. \end{aligned}$$

Here, $k_{||}^2 = k_x^2 + k_y^2$. Since the experimentally measured Luttinger parameters are those of the 6-band k$\cdot$p model, they must be modified to include the electron- and hole-band interactions of the 8-band model^35^. The parameters are therefore transformed as $\gamma _1\xrightarrow {\phantom{a}}\gamma _1 - E_p/\big (3E_g^{\Gamma }\big )$, $\gamma _{2,3}\xrightarrow {\phantom{a}}\gamma _{2,3} - E_p/\big (6E_g^{\Gamma }\big )$ where $E_P = 2m_oP/\hbar ^2$ is the Kane matrix element, *P*, in units of energy and $E^{{\Gamma }}_g$ is the bandgap at the $\Gamma$-point. The term $S_C$ describing the conduction band non-parabolicity must also be modified and is given as $s_C = 1/m_{CB}^* - \big (E_p/3\big )\big [2/E_g^{\Gamma } + 1/\big (E_g^{\Gamma }+\Delta _{so}\big )\big ]$, where $\Delta _{so}$ is the spin-orbit splitting energy. The effect of small residual strains in the bulk material is included using the Bir-Pikus Hamiltonian, resulting in correction terms of^36^

$$\begin{aligned}{} & {} E_{CB}^{GeSn}({\textbf {k}} )\xrightarrow {\phantom{a}} E_{CB}^{GeSn}({\textbf {k}}) + \delta E_{cb}^{hy}, \\{} & {} E_{HH}^{GeSn}({\textbf {k}} ) \xrightarrow {\phantom{a}} E_{HH}^{GeSn}({\textbf {k}}) + \delta E_{vb}^{hy} - \eta _{ax}, \\{} & {} E_{LH}^{GeSn}({\textbf {k}} ) \xrightarrow {\phantom{a}} E_{LH}^{GeSn}({\textbf {k}}) + \delta E_{vb}^{hy} + \eta _{ax}, \\{} & {} E_{SO}^{GeSn}({\textbf {k}}) \xrightarrow {\phantom{a}} E_{SO}^{GeSn}({\textbf {k}}) + \delta E_{vb}^{hy} + \eta _{ax}, \end{aligned}$$

6 $$\begin{aligned} Q({\textbf {k}} ) \xrightarrow {\phantom{a}} Q({\textbf {k}}) - \sqrt{2}\eta _{ax}, \end{aligned}$$

where $\delta E_{cb}^{hy} =-2a_c(1-c_{12}/c_{11})\epsilon _{||}$, $\delta E_{vb}^{hy} =-2a_v(1-c_{12}/c_{11})\epsilon _{||}$ and $\eta _{ax} = -b_{ax}(1+2c_{12}/c_{11})\epsilon _{||}$. $c_{11(12)}$ are the elastic constants, $a_{c(v)}$ is the conduction (valence) band deformation potential, $b_{ax}$ is the axial deformation potential and $\epsilon _{||}$ is the in-plane strain.

From the calculated bands, the material gain at a given photon energy is modelled by summing over the direct transitions between the conduction and valence bands, and integrating over the Brillouin zone using^37^

7 $$\begin{aligned} G(\hbar \omega ) = \frac{\pi q^2}{n_rc\epsilon _0m_0^2\omega } \sum _{c,v} \iiint \frac{\textrm{d}k_x\textrm{d}k_y\textrm{d}k_z}{(2\pi )^3} |\textbf{e}\cdot {\varvec{P}}_{cv}|^2 \times \delta (E_{c} -E_{v} - \hbar \omega )(f_c-f_v), \end{aligned}$$

where the limits are chosen such that the gain is suitably converged. Here, *q* is the electron charge, $n_r$ is the wavelength-dependent refractive index, *c* is the speed of light, $\epsilon _0$ is the vacuum permittivity, $m_0$ is the electron rest mass, $\omega$ is the photon angular frequency and $|\textbf{e}\cdot {\varvec{P}}_{cv}|^2$ is the momentum matrix element, calculated from the eigenvectors of the k$\cdot$p Hamiltonian. In practice, the idealised delta function is replaced by a Lorentzian lineshape (with a half-width at half-maximum of $\sigma =\hbar /\tau$) to account for inherent broadening from finite carrier lifetimes and scattering. Due to a lack of literature values, an inverse relation between the temperature and carrier lifetime of $\tau = 100~\textrm{fs}\times 300~\textrm{K}/T$ is adopted based on similar observations in quantum well devices^38^. However, the effect of this choice on the qualitative trends discussed in this work is negligible. The thermal distribution of electrons at quasi-equilibrium is described by the Fermi-Dirac distribution f$_\mathrm {c(v)}$, which is given by

8 $$\begin{aligned} f_{c(v)} = \bigg [1+\textrm{exp}\bigg (\frac{E_{c(v)}(k_x,k_y,k_z)-E_{fc(v)}}{k_BT}\bigg )\bigg ]^{-1}, \end{aligned}$$

where $E_{fc(v)}$ are the conduction (valence) quasi-Fermi levels, which describe the electron and hole populations in their respective bands. The quasi-Fermi levels are calculated by iterating an initial trial value until the carrier densities in the conduction and valence bands,

9 $$\begin{aligned} n_{3D} = \sum _i^c \iiint \frac{\textrm{d}k_x\textrm{d}k_y\textrm{d}k_z}{(2\pi )^3}\bigg [1+\textrm{exp}\bigg (\frac{E_i^ \Gamma (k_x,k_y,k_z)-E_{fc}}{k_BT}\bigg )\bigg ]^{-1} + 8\bigg (\frac{m_{L}^{dos}k_BT}{2\pi \hbar ^2}\bigg )^{3/2}F_{1/2}\bigg (\frac{E_{CB}^{L}-E_{fc}}{k_BT}\bigg ) \end{aligned}$$

and

10 $$\begin{aligned} p_{3D} = \sum _i^v \iiint \frac{\textrm{d}k_x\textrm{d}k_y\textrm{d}k_z}{(2\pi )^3}\bigg [1+\textrm{exp} \bigg (\frac{E_{fv}-E_i^v(k_x,k_y,k_z)}{k_BT}\bigg )\bigg ]^{-1}, \end{aligned}$$

respectively, converge to the chosen injected carrier density. The first term in Eqn. 9 denotes the carrier density of the conduction band states around the $\Gamma$-point determined from the k$\cdot$p band structure, while the second term accounts for the inclusion of the L-valley, which is treated as parabolic. Here $m_L^{dos}$ is the density of states effective mass for the L-valley and $F_{1/2}$ is the Fermi-Dirac integral for which the treatment of Fukushima is invoked to ensure a high degree of accuracy for all levels of injection, whilst maintaining fast calculations^39^.

By calculating the gain spectra as a function of carrier injection and temperature using Eqs. (7)–(10), the temperature dependence of the transparency carrier density was obtained. This value constitutes the minimum carrier density required to satisfy the Bernard-Duraffourg condition, above which lasing would be achieved in the absence of optical losses, i.e. $n_{th}=n_{tr}$^40^. The quasi-Fermi levels at which transparency is achieved were then used to calculate the carrier densities in each valley. As such, this is a useful metric for determining the influence of purely band structure related effects on device operating temperature.

Figure 3b depicts the transparency carrier density as a function of temperature for Ge$\mathrm {_{0.895}Sn_{0.105}}$ with a small residual compressive strain of −0.27% (red), as per the experimentally measured devices^41^, which is compared to unstrained (black), and 0.27% tensile strained material (blue). At temperatures around 50 K, there is a factor of ~2 improvement in the transparency carrier density for the strained materials. This stems from the lifting of light- and heavy-hole band degeneracy, resulting in greater penetration of the hole quasi-Fermi level into the valence band for a given carrier injection. However, the compressive strain also results in an appreciable reduction in the ${\Gamma }$-L splitting. As such, above ~100 K, the enhanced population in the L-valley due to thermal broadening offsets the benefits afforded by the reduced valence band density of states, resulting in a higher transparency carrier density for the compressively strained material. In contrast, small amounts of residual tensile strain can result in a marginal improvement in the temperature dependence of the transparency carrier density. The resulting change in the bulk density of states is comparable to the compressively strained case^42^, however the material directness is increased. At higher temperatures, these effects compound such that high temperature performance is better than both compressive and unstrained material. As noted by Thai *et al.* the inclusion of tensile strain also reduces optical loss through intervalence band absorption due to enhanced splitting between the light-hole and spin-orbit bands, leading to higher material gain for a given carrier injection^43^. Additionally, as the light-hole band is predominantly formed from z-oriented p-orbitals, there is enhanced emission into the TM mode which is preferable for edge emitting lasers.

As such it is important to relieve even small levels of compressive strain which can act to lower the operational temperature by decreasing the active region directness. In bulk structures, only very low levels of residual tensile strain would be attainable and would require growth on virtual substrates such as SiGeSn with higher levels of Sn than the active region. This would likely hinder performance further due to the increase in defect density and less favourable band alignments. Thus, the only viable option for significantly improving directness in bulk heterostructure devices is to alter the active material composition.

**Figure 4:**
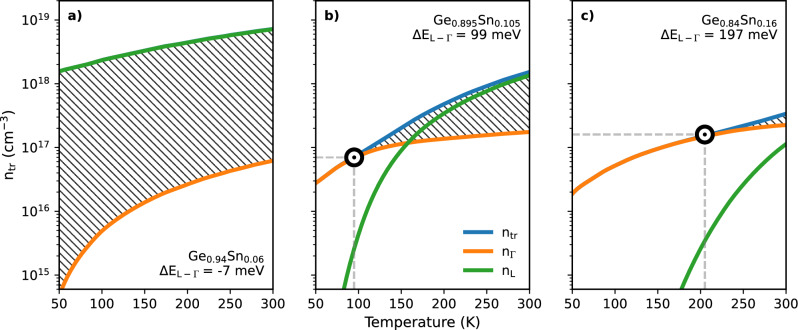
The carrier density as a function of temperature required to reach transparency for (**a**) 6%, (**b**) 10.5% and (**c**) 16% Sn unstrained material. Blue lines denote the total transparency carrier density, whilst the green and orange lines are the distribution of those carrier densities between the L and $\Gamma$-valleys. Markers denote the temperature and carrier density at which carriers in the L-valley account for 2% of threshold and we therefore deem to become ‘significant’. Levels of directness are quoted for T = 300 K.

For Sn fractions <~10% the band structure exhibits strong alloy-induced band-mixing effects which limits the applicability of the ‘direct’ and ‘indirect’ labels^7^. At 6% Sn, the fractional L-character of the conduction band edge is significant, resulting in transparency carrier densities in excess of $\mathrm {8\times 10^{18}~cm^{-3}}$ at RT, with over 99% of injected carriers occupying L-valley like states, as shown in Fig. 4a. The necessity for such high carrier densities in the absence of optical losses renders GeSn with low Sn fractions impractical for bulk device applications.

At 10.5% Sn, corresponding to the experimentally investigated device, the transparency carrier density is greatly reduced for all temperatures. This stems from increased directness which enables carriers to occupy states in the $\Gamma$-valley at low temperatures. As temperature increases, thermal broadening of the carrier distribution results in heightened L-valley occupation, drastically raising the transparency carrier density. At RT the transparency carrier density is calculated as ~2${\times {10}^{18}~\textrm{cm}^{-3}}$, as per Fig. 4b, which is comparable to standard III-V materials such as GaAs, despite high carrier concentrations in the L-valley^44^. This is the culmination of two distinct processes. Firstly, the density-of-states in the $\Gamma$-valley of GeSn is approximately a third of that in GaAs, which means fewer carriers are required to achieve population inversion in bulk GeSn. However, due to low levels of directness, the fraction of injected carriers occupying the $\Gamma$-valley is less than 10% at RT. This counters the benefits of the smaller direct valley effective mass, resulting in similar thresholds for material gain when compared to standard III-V semiconductors. In practice, the necessity to inject a higher overall carrier density in order to populate direct $\Gamma$-states and achieve material gain would raise the lattice temperature, and thus further increase the number of carriers occupying indirect L-states. With no radiative recombination paths available for carriers in the L-valleys, optical losses would be large due to a thermal runaway of FCA, significantly raising the threshold current and degrading device performance^32^. Thus, FCA from L-valley carriers in combination with other loss processes such as via defect-related recombination likely limit the maximum operational temperature by significantly increasing the threshold current density.

As shown in Fig. 4c, the effect of L-valley occupation on transparency can be reduced by further increasing the Sn concentration. This is illustrated by the shift in the break point, defined here as the temperature at which the L-valley occupation fraction is 2% at threshold, shown by white markers in figures b and c. However, around 30% of the injected carriers at threshold occupy L-valley states at RT for 16% Sn. By extrapolation, it is predicted that around 20% Sn would be required to minimise this, whilst remaining within experimentally feasible Sn compositions using CVD growth. Such high incorporation has been achieved in optically pumped devices which elicit near RT operation^10^. It is likely that this will require significant development in growth techniques to integrate such high Sn content into a device heterostructure, and ensure adequate band edge offsets and material quality to produce working, electrically pumped devices.

### Auger resonances

**Figure 5:**
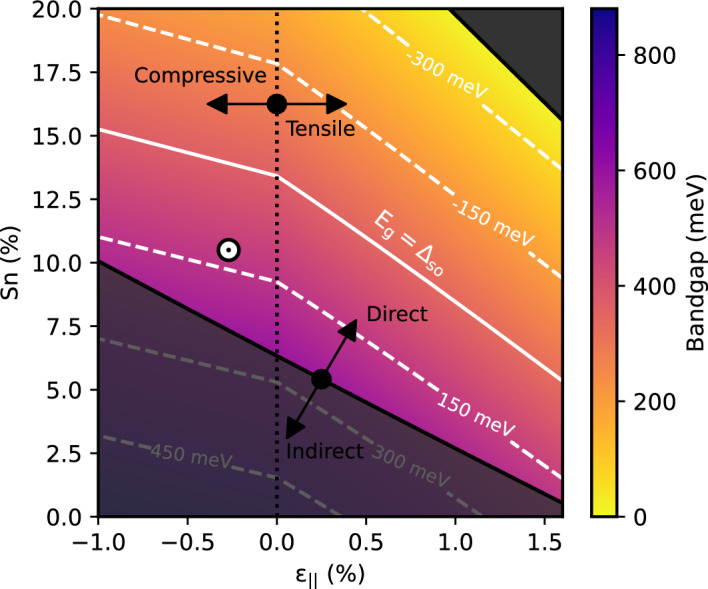
Bandgap as a function of Sn content and in-plane strain. Indirect compositions are greyed, whilst material with high tensile strain and large Sn concentrations that produce negative bandgap material are neglected. The coloured region therefore represents the composition space in which direct material can be achieved. The white marker illustrates the devices investigated in this work. White dashed lines depict $E_g-\Delta _{SO}$.

Although increasing tensile strain and Sn content offer routes to improve directness, the effect of the incurred band structure changes on other carrier recombination mechanisms must be carefully considered. For example, Fig. 5 shows the bandgap as a function of Sn content and in-plane strain, $\varepsilon _{\parallel }$, at 300 K. The white lines depict the difference between the bandgap and spin-orbit splitting energies. When this splitting is small, carriers close to the zone centre are able to undergo non-radiative CHSH Auger recombination, whereby the energy of a recombining Conduction band electron and Heavy hole is transferred to an electron in the Spin-orbit band which is excited into the Heavy hole band, a process found to be problematic in III-V based telecoms lasers^45^. This will lead to a so-called “CHSH resonance” when the two energies become equal^46^. The CHSH Auger rate is proportional to p$^2$n$_{\Gamma }$, where p is the hole density in the VB and n$_\Gamma$ is the electron density in the $\Gamma$-valley. Since the transparency carrier density has been shown to be high for alloy compositions close to this resonance, the hole density will be large, likely resulting in significant non-radiative recombination. Such recombination leads to device heating and the large temperature sensitivity of this process may act to further inhibit high-temperature operation. Hence, whilst directness may be improved by increasing tensile strain and/or Sn content, there is a performance trade-off due to enhanced CHSH Auger recombination which must be considered during the design stage.

Incorporating a significant amount of Sn and/or tensile strain can tune the device through the CHSH resonance and provide sufficient energetic separation to suppress CHSH Auger processes at the cost of a greatly reduced bandgap. Pushing emission further into the mid-infrared in this manner results in an increase in hot electron producing CHCC Auger recombination, where the energy from an interband transition is used to promote an electron to a higher conduction band state. This occurs because energy and momentum conservation laws can be satisfied by carriers closer to the zone centre as the bandgap is reduced. For this reason, idealised bulk devices would contain an active region with Sn concentration of ~20%, allowing for significant improvements in the directness, whilst also tuning the band structure beyond the CHSH Auger resonance and limiting CHCC recombination.

## Discussion

An electrically pumped, quantum confined laser is a highly desirable next step due to the promise of reduced threshold currents, and wavelength tunability through the choice of layer thickness. However, in current heterostructure designs, the use of thin GeSn quantum wells is impractical at low Sn concentrations as confinement results in decreased material directness^33^. Thus, for the realisation of quantum confined, electrically injected devices sustained efforts must be focused into three distinct areas.

The first of which is to improve the growth quality of high Sn material, which is key to reduce trap levels that can act as non-radiative recombination centres, as observed in the bulk devices in this study. Recently, aspect ratio trapping (ART) growth has been proposed as a selective area growth technique with the potential to address this. ART acts to confine {111} oriented defects such as threading dislocations by beginning growth in a tall, narrow trench etched into a dielectric mask. This results in a termination of this type of defect at the trench walls, greatly reducing defectivity in the epilayers. In 2023, De Koninck et al. utilised ART to demonstrate electrically injected GaAs-based nanoridge lasers with impressive performance metrics at RT^47^. However, growth with group-IV material remains preferable for CMOS integration due to better thermal budget, no anti-phase domain issue and the potential for ion-implantation. Since the technique inherently enables relaxed growth, it may also enable higher Sn concentrations and greater layer uniformity, previously hindered by compressive strains, to be achieved^48^. Thus, thin, high quality GeSn-based quantum wells can be envisaged with sufficient levels of directness to achieve lasing.

The second approach is to maximise the heterostructure design space, for example through focused investigations of new group-IV materials such as dilute carbides^49^, owing to their predicted direct bandgap. Recent theoretical studies of carbide-based quantum wells indicate that high levels of directness can persist even in thin wells, in contrast to GeSn^50^. The study suggests that Ge is then a suitable barrier material, and SiGeSn can be freed for use in the separate confinement layers to improve optical confinement. However, another group have suggested that Ge:C is only quasi-direct, owing to the observed conduction band minima at $\Gamma$ primarily having the L-like character of the Ge host matrix, making it optically inactive^51^. Thus, further experimental investigations of group-IV alloys are required in order to broaden the options available during the design process.

Finally, improvements to the design process itself will enable the accurate design and optimisation of structures prior to fabrication. Given the large scatter in reported parameters observed in the literature, specifically in the aforementioned bowing parameters, the determination and standardisation of material parameters for group-IV alloys is critical for further theoretical design work to be effectively carried out.

## Conclusion

In conclusion, we have experimentally demonstrated that appreciable levels of carrier leakage to the L-valley are observed in electrically injected, bulk GeSn double heterostructures with 10.5% Sn. This constitutes ~1% of the threshold current at 85 K and limits their maximum operational temperature. A large fractional contribution from a pressure-insensitive process was found, which we attribute to defect-related recombination in the GeSn buffer due to the high level of threading dislocations. Semi-empirical calculations of the bulk material gain allowed for the investigation of the transparency carrier density as a function of temperature. These results show that, even for the idealised case, there is significant occupation of L-valley states at low temperature leading to a large population of carriers able to contribute to FCA losses. Thus, the Sn composition should be increased towards 20% in bulk devices to achieve adequate confinement at RT and ensure the CHSH resonance is avoided. In practice, electrically injected, quantum confined devices are a highly desirable next step from bulk devices, however further work is required to produce feasible designs, potentially through the use of new group-IV alloys or utilising the ART growth technique.

## Data Availability

The datasets generated during and/or analysed during the current study are available from the corresponding author on reasonable request.
